# Carbon Nanotubes: Applications in Pharmacy and Medicine

**DOI:** 10.1155/2013/578290

**Published:** 2013-09-30

**Authors:** Hua He, Lien Ai Pham-Huy, Pierre Dramou, Deli Xiao, Pengli Zuo, Chuong Pham-Huy

**Affiliations:** ^1^China Pharmaceutical University, Nanjing 210009, China; ^2^Key Laboratory of Drug Quality Control and Pharmacovigilance, Ministry of Education, China Pharmaceutical University, Nanjing 210009, China; ^3^Department of Pharmacy, Stanford University Medical Center, Palo Alto, CA 94304, USA; ^4^Faculty of Pharmacy, University of Paris V, 4 avenue de l'Observatoire, 75006 Paris, France

## Abstract

Carbon nanotubes (CNTs) are allotropes of carbon, made of graphite and constructed in cylindrical tubes with nanometer in diameter and several millimeters in length. Their impressive structural, mechanical, and electronic properties are due to their small size and mass, their strong mechanical potency, and their high electrical and thermal conductivity. CNTs have been successfully applied in pharmacy and medicine due to their high surface area that is capable of adsorbing or conjugating with a wide variety of therapeutic and diagnostic agents (drugs, genes, vaccines, antibodies, biosensors, etc.). They have been first proven to be an excellent vehicle for drug delivery directly into cells without metabolism by the body. Then other applications of CNTs have been extensively performed not only for drug and gene therapies but also for tissue regeneration, biosensor diagnosis, enantiomer separation of chiral drugs, extraction and analysis of drugs and pollutants. Moreover, CNTs have been recently revealed as a promising antioxidant. This minireview focuses the applications of CNTs in all fields of pharmacy and medicine from therapeutics to analysis and diagnosis as cited above. It also examines the pharmacokinetics, metabolism and toxicity of different forms of CNTs and discusses the perspectives, the advantages and the obstacles of this promising bionanotechnology in the future.

## 1. Introduction

Carbon nanotubes (CNTs), discovered by Japanese scientist Iijima in 1991 [1], are now considered to be a top class subject in academic researches as well as in various industrial areas. These nanomaterials are allotropes of carbon, made of graphite, and have been constructed in cylindrical tubes with nanometer scale in diameter and several millimeters in length [2, 3]. Their impressive structural, mechanical, and electronic properties are due to their small size and mass, their incredible mechanical strength, and their high electrical and thermal conductivity [4, 5]. Carbon nanotubes have been first used as additives to various structural materials for electronics, optics, plastics, and other materials of nanotechnology fields. Since the beginning of the 21st century, they have been introduced in pharmacy and medicine for drug delivery system in therapeutics. Thanks to their high surface area, excellent chemical stability, and rich electronic polyaromatic structure, CNTs are able to adsorb or conjugate with a wide variety of therapeutic molecules (drugs, proteins, antibodies, DNA, enzymes, etc.). They have been proven to be an excellent vehicle for drug delivery by penetrating into the cells directly and keeping the drug intact without metabolism during transport in the body [2–5]. Many studies have demonstrated that when bonded to CNTs, these molecules are delivered more effectively and safely into cells than by traditional methods [3–5]. This fantastic discovery has opened a new way for drug preparations that is completely different with traditional techniques used in pharmaceutical industry before and radically changed anterior concepts of pharmacology [3, 4]. It has been first applied to bind antineoplastic and antibiotic drugs to carbon nanotubes for cancer and infection treatments, respectively. Then, other linkages of biomolecules (genes, proteins, DNA, antibodies, vaccines, biosensors, cells, etc.) to CNTs havee been also assayed for gene therapy, immunotherapy, tissue regeneration and diagnosis of different ailments [6–10]. Therefore, in a very short time, CNTs have become the focus of attention by scientists in a wide variety of disciplines. They may be promising antioxidants for health protective effect and ailment prevention in the future [11]. However, it is notified that all these medicinal findings are in an experimental stage and are still not applied in men. Besides these main applications of CNTs, they have been shown as a powerful tool for enantiomer separation of chiral drugs and chemicals in pharmaceutical industry as well as in laboratory and for extraction of drugs and pollutants in different media by solid phase extraction before analysis [12, 13]. Our group has recently contributed to the development of different novel functionalized CNT techniques for drug delivery as well as for drug analysis and also to the study on the interactions between CNTs and albumin or drugs [14–23]. 

In this paper, an overview of different applications of CNTs is focused in the field of pharmacy and medicine. It briefly describes the structure of CNTs and their different types. It examines some main methodologies using CNTs as vehicle for drug and biomolecule delivery in the treatment and diagnosis of different dreadful diseases. It enumerates some recent researches of CNTs as antioxidants and also some novel analytical techniques using CNTs as tools for enantioseparation of chiral drugs and for solid phase extraction of drugs and pollutants in different media. Pharmacokinetics, metabolism, and toxicity of CNTs are also examined. The perspectives of this promising bionanotechnology in the future medicine are briefly commented, in the conclusion.

## 2. Carbon Nanotubes: Structures, Types and Preparation

**Figure 1:**
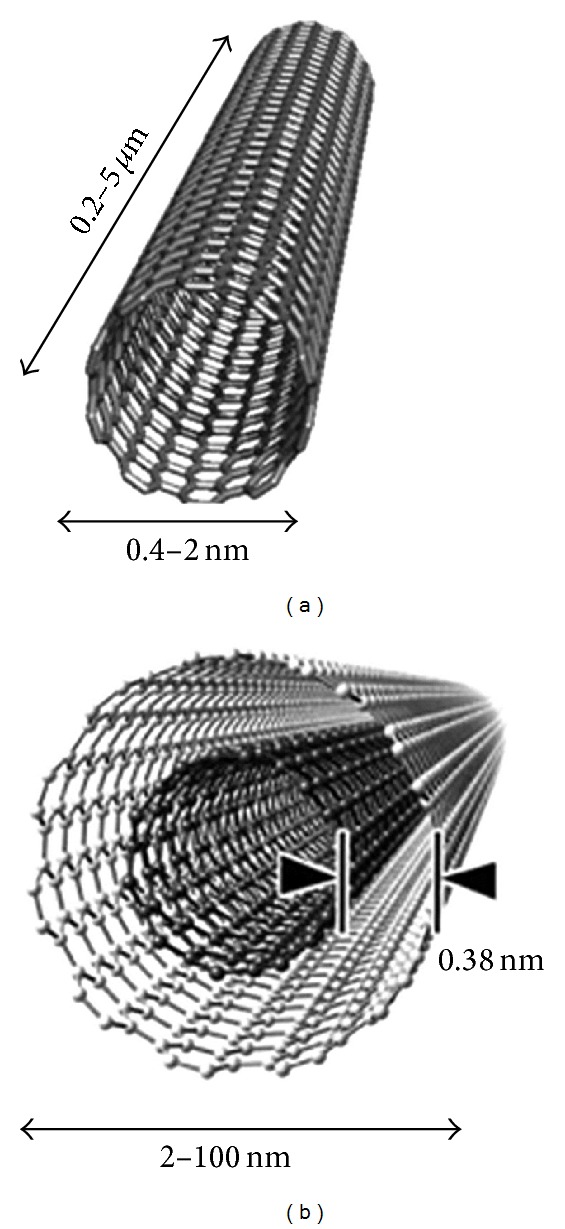
Conceptual diagrams of single-walled carbon nanotubes (SWCNT) (a) and multi-walled carbon nanotubes (MWCNT) (b). Image from free internet access.

Carbon nanotubes (CNTs) consist exclusively of carbon atoms arranged in a series of condensed benzene rings rolled up into a tubular structure. This novel artificial nanomaterial belongs to the family of fullerenes, the third allotropic form of carbon along with graphite and diamond which are both natural sp2 (planar) and sp3 (cubic) forms, respectively [2, 3, 7]. Based on the number of layers, structures of CNTs are classified into two types: single-walled carbon nanotubes (SWCNTs) and multiwalled carbon nanotubes (MWCNTs) (Figure 1).

**Figure 2:**
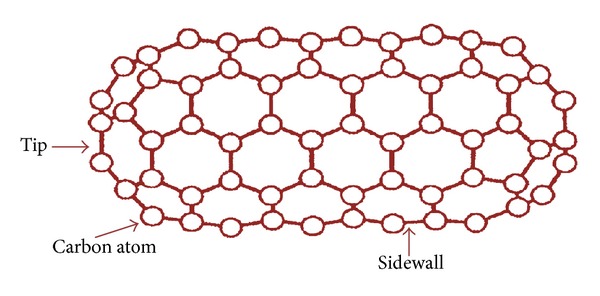
A carbon nanotube with closed ends.

**Figure 3:**
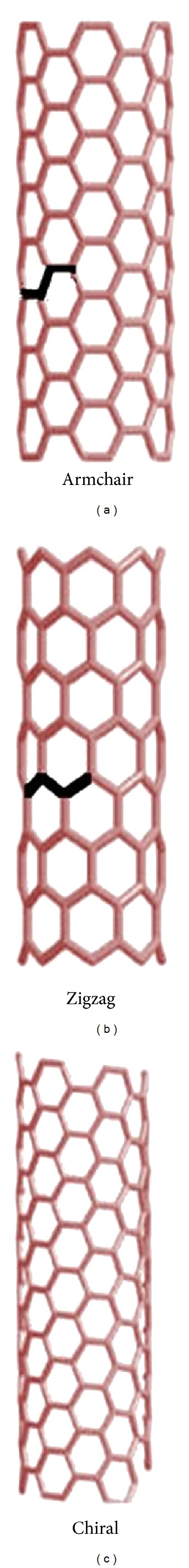
Carbon nanotube structures of armchair, zigzag and chiral configurations. They differ in chiral angle and diameter: armchair carbon nanotubes share electrical properties similar to metals. The zigzag and chiral carbon nanotubes possess electrical properties similar to semiconductors.

SWCNTs consist of a single graphene cylinder with diameter varying between 0.4 and 2 nm, and usually occur as hexagonal close-packed bundles (Figure 2). MWCNTs consist of two to several coaxial cylinders, each made of a single graphene sheet surrounding a hollow core. The outer diameter of MWCNTs ranges from 2 to 100 nm, while the inner diameter is in the range of 1–3 nm, and their length is 0.2 to several *μ*m [3, 10]. From a chemical reactivity point of view, CNT can be differentiated into two zones: the tips and the sidewalls. An important factor that controls these unique properties comes from a variation of tubule structures that are caused by the rolling up of the graphene sheet into a tube. There are three distinct ways for the molecule to do the rolling, depending upon its direction: armchair, zigzag, and chiral (Figure 3). Detailed explanations of CNTs structures can be found in several recent review articles cited herein [3–7, 24–27]. Structures and characterizations of SWCNTs and MWCNTs are summarized in Table 1 according to Singh et al. [3].

Three main techniques generally used for SWCNTs and MWCNTs production are: Arc-Discharge method (using arc-vaporization of two carbon rods), Laser Ablation method (using graphite), and Chemical Vapor Deposition (using hydrocarbon sources: CO, methane, ethylene, acetylene). After preparation, CNTs are submitted to purification by acid refluxing, surfactant aided sonication, or air oxidation procedure in order to eliminate impurities such as amorphous carbon, fullerenes, and transition metals introduced as catalysts during the synthesis [3, 4, 26]. Pristine CNTs are now synthesized and marketed by many chemical firms worldwide. 

## 3. Carbon Nanotubes: Functionalization for Biomedical Applications

Pristine CNTs are not soluble in aqueous solutions because they have highly hydrophobic surfaces. Surface functionalization is required to solubilize CNTs, and to render biocompatibility and low toxicity for their medical applications [25]. The functionalization procedure of CNTs can be divided into two main approaches, depending on the nature of the biomolecule linked to carbon nanotube, that is, covalent attachment (chemical bond formation) and noncovalent attachment (physioadsorption) [5, 27].

**Figure 4:**
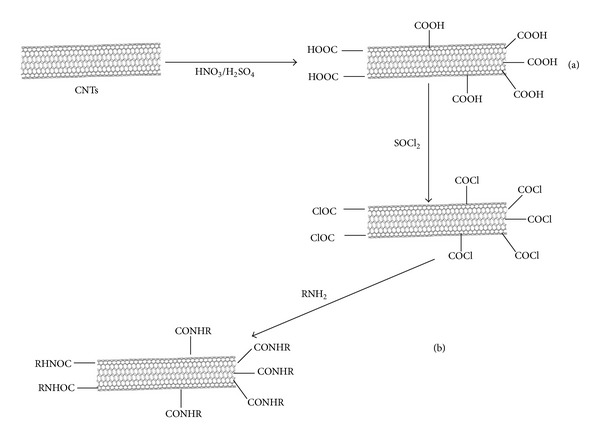
Covalent functionalization of CNTs by (a) oxidation reaction by strong acid and (b) further attaching hydrophilic molecules by amidation reactions.

The covalent functionalization of CNTs is generally obtained by oxidation with strong acids (HNO_3_). During the process, carboxyl (–COOH) groups are formed at the open sides (tips) and at the defects on the sidewalls of SWCNT or MWCNT, then, further covalent conjugation with amino acid. For the creation of –COOH on the sidewalls of CNTs, nitrene cycloaddition, arylation using diazonium salts or 1,3-dipolar cycloadditions are usually employed [5–7, 27]. Schematic illustration of covalent functionalization of carbon nanotubes is summarized in Figure 4.

The noncovalent functionalization of CNTs can be carried out by coating CNTs with amphiphilic surfactant molecules or polymers (polyethyleneglycol). The large aromatic (*π*-electrons) hydrophobic surface of carbon nanotubes makes them ideal partners for noncovalent interactions with suitable complementary molecules and macrobiomolecules (DNA). These interactions can take place both on the inside and outside of CNTs. However, macromolecules cannot be linked on their inside [5, 26, 27]. 

**Figure 5:**
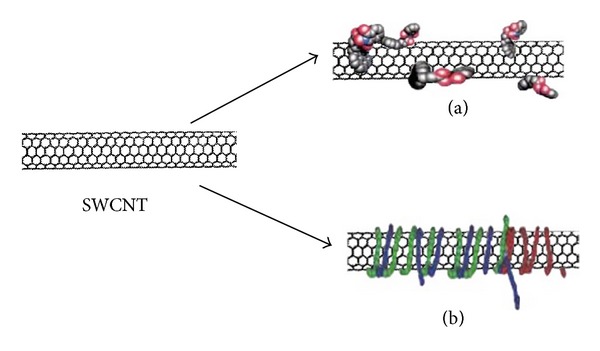
Noncovalent functionalization of CNTs with (a) surfactants such as protein adsorption and (b) polymers such as DNA wrapping.

Schematic noncovalent functionalization of CNTs is illustrated in Figure 5.

After functionalization, CNTs become hydrophilic and are ready to be linked with drugs or biomolecules (genes, DNA, proteins, enzymes, biosensors, etc.) for their delivery into the target cells or organs. Detailed techniques of functionalization and linkage can be found in our recent publications and in the others cited herein [4–7, 18–20, 23, 27]. 

## 4. Applications of Carbon Nanotubes in Pharmacy and Medicine

**Figure 6:**
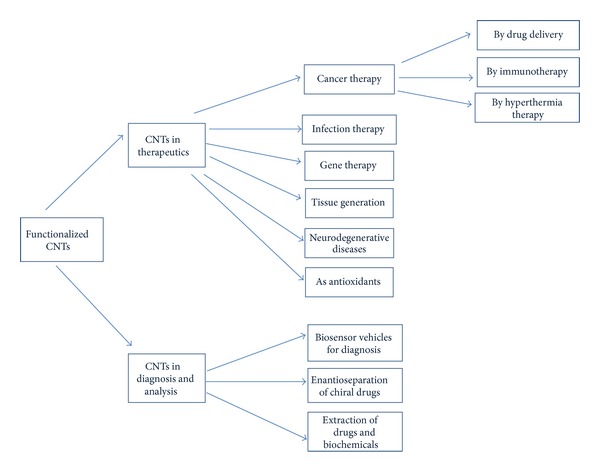
Schematic of carbon nanotube applications in therapeutics and biomedical diagnosis and analysis.

The main applications of CNTs in pharmacy and medicine include drug, biomolecule, gene delivery to cells or organs, tissue regeneration, and biosensor diagnostics and analysis. They are summarized in Figure 6.

**Figure 7:**
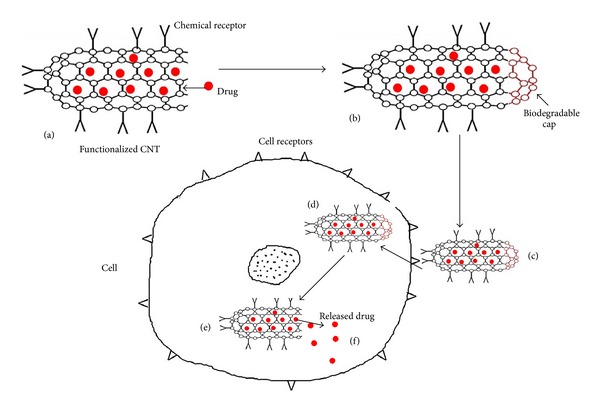
Schematic illustration of the drug delivery process. (a) CNT surface is linked with a chemical receptor (Y) and drugs (●) are loaded inside, (b) open end of CNT is capped, (c) drug-CNT carrier is introduced in the body and reaches the target cells due to chemical receptor on CNT surface, (d) cell internalizes CNT by cell receptors (V) via endocytosis pathway for example, (e) cap is removed or biodegrades inside the cell, then drugs are released.

For drug delivery, the general process using CNTs can be briefly resumed as follows. Drug is fixed on the surface or the inside of functionalized CNTs. The conjugate obtained is then introduced into the animal body by classic ways (oral, injection) or directly to the target site through the use of a magnetic conjugate, for example, guided by an external magnet to the target organ, such as lymphatic nodes. The cell ingests the drug CNT capsule and finally the nanotube spills its contents into the cell and thus the drug is delivered [3–8]. Schematic illustration of drug delivery process is described in Figure 7.

Generally, functionalized CNTs possess the ability to carry molecules of interest across the cytoplasmic membrane and nuclear membrane without producing a toxic effect; therefore, the drug CNT conjugate proves to be safer and more effective than drug used alone by traditional preparation. After reaching the targeted cell, they are two possibilities to deliver the drug: the drug enters the cells without internalization of the CNT carrier or both the drug and the CNT carrier enters the cells. The latter internalization method is more effiectiv than the first one, because after entering the cells, the intracellular environment degrades the drug carrier conjugate releasing drug molecules in situ, that is, inside the cells. While, in the non-internalization method, the extracellular environment helps to degrade drug carrier conjugates and the drug then crosses itself the lipid membrane to enter the cells, thereby, there is a possibility of drug degradation during this penetration by itself. There are two possible mechanisms of CNT internalization: either via the endocytosis pathway or via the insertion and diffusion pathway, that is, via the endocytosis-independent pathway. However, the mechanism of the last pathway is still not well-known [5, 7, 27]. 

Briefly, the explanations about the ability of CNTs to cross cell membranes for drug delivery are due to the simple hydrophobic interaction, *π*-*π* stacking interaction, electrostatic adsorption and covalent bonds in their structure, but also by adsorption into the hollow cylinder, which is conducive to increase the adsorptive capacity [20]. Besides, CNTs have a capacity not only to penetrate into the cells to promote the cellular uptake of therapeutic molecules but also to keep them intact during transportation and cellular penetration. The last property permits the decrease of the dosages of drugs used, and consequently their toxicity, especially for anticancer drugs. 

### 4.1. Carbon Nanotubes Used for Cancer Therapy

#### 4.1.1. By Drug Delivery

CNTs can be used as drug carriers to treat tumors [8, 24–31]. The efficacy of anticancer drugs used alone is restrained not only by their systemic toxicity and narrow therapeutic window but also by drug resistance and limited cellular penetration. Because CNTs can easily across the cytoplasmic membrane and nuclear membrane, anticancer drug transported by this vehicle will be liberated in situ with intact concentration and consequently, its action in the tumor cell will be higher than that administered alone by traditional therapy. Thus, the development of efficient delivery systems with the ability to enhance cellular uptake of existing potent drugs is needed. The high aspect ratio of CNTs offers great advantages over the existing delivery vectors, because the high surface area provides multiple attachment sites for drugs [18]. 

Many anticancer drugs have been conjugated with functionalized CNTs and successfully tested in vitro and in vivo such as epirubicin, doxorubicin, cisplatin, methotrexate, quercetin, and paclitaxel [18, 23, 29–32].

For avoiding the harmful effect of anticancer drug on healthy organs and cells, our group [23] has linked epirubicin with a magnetic CNTs complex obtained by fixing a layer of magnetite (Fe_3_O_4_) nanoparticles on the surface of the nanotubes with necklace-like type and on the tips of shortened MWCNTs [23]. Other authors have used the epirubicin magnetic CNTs complex for lymphatic tumor targeting. Such a system can be guided by an externally placed magnet to target regional lymphatic nodes [8]. 

For the same previous reason, chemotherapeutic agents can be bound to a complex formed by CNT and antibody against antigen overexpressed on the cancerous cell surface. By the attraction of antigen-antibody, the CNTs can be taken up by the tumor cell only before the anticancer drug is cleaved off CNTs; thus, targeting delivery is realized [29–31]. A major obstacle to effective anticancer therapy is the multidrug resistance caused by enhanced efflux of anticancer drugs by the overexpressed p-glycoprotein, resulting in poor anticancer effect [31]. Li and coworkers [32] have shown that SWCNTs can be functionalized with p-glycoprotein antibodies and loaded with the anticancer agent doxorubicin. Compared with free doxorubicin, this formulation demonstrated higher cytotoxicity by 2.4-fold against K562R leukemia cells.

The in vivo administration of SWCNT paclitaxel conjugate in a murine breast cancer model has been observed with higher efficacy in suppressing tumor growth and less toxic effects to normal organs [8, 29]. The higher therapeutic efficacy and lower side effects could be attributed to prolonged blood circulation, higher tumor uptake, and slower release of drug from SWCNTs [26].

#### 4.1.2. By Antitumor Immunotherapy

Some studies have demonstrated that CNTs used as carriers can be effectively applied in antitumor immunotherapy [3, 26–33]. This therapeutic consists of stimulating the patient's immune system to attack the malignant tumor cells. This stimulation can be achieved by the administration of a cancer vaccine or a therapeutic antibody as drug. Some authors have validated the use of CNTs as vaccine delivery tools [33]. Yang's group observed that the conjugate of MWCNTs and tumor lysate protein (tumor cell vaccine) can considerably and specifically enhance the efficacy of antitumor immunotherapy in a mouse model bearing the H22 liver tumor [27]. In vitro, the conjugate of CNTs and tumor immunogens can act as natural antigen presenting cells (such as mature dendritic cells) by bringing tumor antigens to immune effector T cells; this action is due to the high avidity of antigen on the surface and the negative charge. The complement system activation effects of CNTs and also their adjuvant effects may play a role in the stimulation of antitumor immunotherapy; however, the mechanism remains unknown [27, 33]. 

#### 4.1.3. By Local Antitumor Hyperthermia Therapy

The hyperthermia therapy using CNTs has been recently suggested as an efficient strategy for the cancer treatments. SWCNTs exhibit strong absorbance in the near-infrared region (NIR; 700–1100 nm). These nano-materials are considered as potent candidates for hyperthermia therapy since they generate significant amounts of heat upon excitation with NIR light [29–31]. The photothermal effect can induce the local thermal ablation of tumor cells by excessive heating of SWCNTs shackled in tumor cells such as pancreatic cancer. Some progress in the technique has been achieved in recent years, and it has shown feasibility in clinical application.

### 4.2. Carbon Nanotubes for Infection Therapy

Because of the resistance of infectious agents against numerous antiviral, antibacterial drugs or due to certain vaccine inefficacy in the body, CNTs have been assayed to resolve these problems. Functionalized CNTs have been demonstrated to be able to act as carriers for antimicrobial agents such as the antifungal amphotericin B [9, 34]. CNTs can attach covalently to amphotericin B and transport it into mammalian cells. This conjugate has reduced the antifungal toxicity about 40% as compared to the free drug [34]. Our group has successfully combined an antimicrobial agent Pazufloxacin mesilate with amino-MWCNT with high adsorption and will be applied to experimental assays for infection treatment [20]. 

Functionalized CNTs can also act as vaccine delivery procedures [24, 34]. The linkage of a bacterial or viral antigen with CNTs permits of keeping intact antigen conformation, thereby, inducing antibody response with the right specificity [26]. The fixation of functionalized CNTs with B and T cell peptide epitopes can generate a multivalent system able to induce a strong immune response, thereby becoming a good candidate for vaccine delivery [4, 27]. Thus, functionalized CNTs can act a good carrier system for the delivery of candidate vaccine antigens. Besides, CNTs themselves might have antimicrobial activity since bacteria may be adsorbed onto the surfaces of CNTs, such as the case of *E. coli*. The antibacterial effect was attributed to carbon nanotube-induced oxidation of the intracellular antioxidant glutathione, resulting in increased oxidative stress on the bacterial cells and eventual cell death [26].

### 4.3. Carbon Nanotubes for Gene Therapy by DNA Delivery

Gene therapy is an approach to correct a defective gene which is the cause of some chronic or hereditary diseases by introducing DNA molecule into the cell nucleus. Some delivery systems for DNA transfer include liposomes, cationic lipids and nanoparticles such as CNTs recently discovered [10, 24, 28].

When bound to SWCNTs, DNA probes are protected from enzymatic cleavage and interference from nucleic acid binding proteins, consequently, DNA-SWCNT complex exhibits superior biostability and increases self-delivery capability of DNA in comparison to DNA used alone [4, 10, 14]. Indeed, stable complexes between plasmid DNA and cationic CNTs have demonstrated the enhancement of gene therapeutic capacity compared with naked DNA. CNTs conjugated with DNA were found to release DNA before it was destroyed by cells defense system, boosting transfection significantly [7, 22, 24].

The use of CNTs as gene therapy vectors has shown that these engineered structures can effectively transport the genes inside mammalian cells and keep them intact because the CNT-gene complex has conserved the ability to express proteins [14]. Pantarotto and coworkers [33] have developed novel functionalized SWCNT-DNA complexes and reported high DNA expression compared with naked DNA. 

### 4.4. Carbon Nanotubes for Tissue Regeneration and Artificial Implants

The knowledge advances of cell and organ transplantation and of CNT chemistry in recent years have contributed to the sustained development of CNT-based tissue engineering and regenerative medicine. Carbon nanotubes may be the best tissue engineering candidate among numerous other materials such as natural and synthetic polymers for tissue scaffolds since this nanomaterial is biocompatible, resistant to biodegradation, and can be functionalized with biomolecules for enhancing the organ regeneration. In this field, CNTs can be used as additives to reinforce the mechanical strength of tissue scaffolding and conductivity by incorporating with the host's body [4–6, 10, 24, 35].

Indeed, MacDonald et al., [35] have successfully combined a carboxylated SWCNTs with a polymer or collagen (poly-l-lactide or poly-D,L-lactide-co-glycolide) to form a composite nanomaterial used as scaffold in tissue regeneration. Other tissue engineering applications of CNTs concerning cell tracking and labeling, sensing cellular behavior, and enhancing tissue matrices are also studied recently [3–6, 35]. For example, it has been reported that CNTs can effectively enhance bone tissue regenerations in mice and neurogenic cell differentiation by embryonic stem cells in vitro [3, 5]. 

### 4.5. Carbon Nanotubes for Neurodegenerative Diseases and Alzheimer Syndrome

As a promising biomedical material, CNTs have been used in neurosciences [3, 5, 24, 36]. Because of their tiny dimensions and accessible external modifications, CNTs are able to cross the blood-brain barrier by various targeting mechanisms for acting as effective delivery carriers for the target brain. Yang et al. [36] have observed that SWCNTs were successfully used to deliver acetylcholine in mice brains affected by Alzheimer's disease with high safety range. Many other functionalized SWCNTs or MWSCNTs have been used successfully as suitable delivery systems for treating neurodegenerative diseases or brain tumors [10, 24, 26]. Overall, the results of these studies have indicated that conjugates of CNTs with therapeutic molecules have better effects on neuronal growth than drugs used alone. 

### 4.6. Carbon Nanotubes as Antioxidants

The theory of oxygen-free radicals has been known about fifty years ago. However, only within the last two decades, has there been an explosive discovery of their roles in the development of diseases, and also of the health protective effects of antioxidants [37]. Nevertheless, the potential role of CNTs as free-radical scavengers is still an emerging area of research. Some scientists have recently reported that CNTs and in particular carboxylated SWCNTs are antioxidants in nature and may have useful biomedical applications for prevention of chronic ailments, aging, and food preservation [11, 38]. Francisco-Marquez et al. observed that the presence of –COOH groups would increase the free radical scavenging activity of SWCNTs and that carboxylated SWCNTs are at least as good as, or even better, free radical scavengers than their nonfunctionalized partners [39]. Their antioxidant property has been used in anti-aging cosmetics and sunscreen creams to protect skin against free radicals formed by the body or by UV sunlight [3, 26]. More investigations of different CNT forms in the future are needed to develop their precious effect of free radical scavenger for biomedical and environmental applications, since free radicals are well known to be very damaging species [37, 38].

### 4.7. Carbon Nanotubes as Biosensor Vehicles for Diagnostic and Detection

A biosensor is an analytical device, used for the detection of an analyte that combines a biological component with a physicochemical detector. The use of CNTs in biosensing nanotechnology is recent and represents a most exciting application area for therapeutic monitoring and in vitro and in vivo diagnostics. For example, many searchers have coupled CNTs with glucose-oxidase biosensors for blood sugar control in diabetic patient with higher accuracy and simpler manipulation than biosensors used alone [4, 26, 40]. Other CNT-enzyme biosensors such as CNT-based dehydrogenase biosensors or peroxidase and catalase biosensors have also been developed for different therapeutic monitoring and diagnostics [40, 41]. 

For electrical detection of DNA, the assay sensitivity was higher with alkaline phosphatase (ALP) enzyme linked to CNTs than with ALP alone. The sensitivity of the assay using SWCNT-DNA sensor obtained by integration of SWCNTs with single-strand DNAs (ssDNA) was considerably higher than traditional fluorescent and hybridization assays. This CNT-biosensor-linked assay can be modified for antigen detection by using specific antibody-antigen recognition. Thus, it could provide a fast and simple solution for molecular diagnosis in pathologies where molecular markers exist, such as DNA or protein [24, 40].

Moreover, CNTs have been assayed to detect some organophosphoric pesticides by using acetylcholine esterase immobilized on CNT surface with electrochemical detection [26, 40, 41]. Owing to their length scale and unique structure, the use of CNTs as biosensor vehicle is highly recommended to develop sensitive techniques for diagnostics and analyses from the laboratory to the clinic. 

### 4.8. Carbon Nanotubes for Enantioseparation of Chiral Drugs and Biochemical

In pharmaceutical industries, 56% of the drugs currently in use are chiral products and 88% of the last ones are marketed as racemates consisting of an equimolar mixture of two enantiomers [42]. Recently, US Food and Drug Administration (FDA) recommended the assessments of each enantiomer activity for racemic drugs in body and promoted the development of new chiral drugs as single enantiomers [38]. Therefore, a wide range of new technologies for chiral separation has been developed, among them carbon nanotechnology. Silva et al. [43] have recently used a microcolumn packed with SWCNT as chiral selector for separation of carvedilol enantiomers, a *β*-blocker, with fluorescent detection. Yu et al. [12] have developed a chiral stationary phase of MWCNT cross-linked with hydroxypropyl-*β*-cyclodextrin for enantioseparation of racemic clenbuterol, a bronchodilator, with-high-resolution factor. It is also notified that CNTs are chiral forms due to the helical winding of the graphitic rings around the tube axis. However, they might not be effective enantio-specific adsorbents. In contrast, chiral selector modified CNTs have been successfully assayed to separate enantiomers from many racemic drugs [12].

### 4.9. Carbon Nanotubes for Solid Phase Extraction of Drugs and Biochemicals

Due to their strong interaction with other molecules, particularly with those containing benzene rings, CNTs surfaces possess excellent adsorption ability. Non-functionalized or functionalized CNTs have been investigated as Solid phase Extraction (SPE) adsorbents used alone or in conjugation with classical SPE sorbents (C18 silica, XAD-2 copolymer) for the analytical extraction of drugs, pesticides or natural compounds in different media such as biological fluids, drug preparations, environment, plants, animal organs, and so forth [13, 21]. In several comparative studies CNTs exhibit similar or higher adsorption capacity than silica-based sorbents or macroporous resins. Many applications of CNTs in SPE can be found in different recent review articles dealing with general aspect [13, 44]. For examples, many drugs such as benzodiazepines, sulfonamides, non-steroidal anti-inflammatory (NSAI), barbiturates, antidepressants, propranolol, cinchonine and quinine, and so forth, have been extracted by SPE using either SWCNTs or MWCNTs as adsorbents in different matrices cited above, then analyzed by different physicochemical techniques [13]. Recently, our group [21] has developed a novel molecularly imprinted magnetic solid phase extraction materials fixed on magnetic carbon nanotubes as support for the extraction and determination of an antibiotic, gatifloxacin (GTFX), in serum samples coupled with HPLC. The results of this novel adsorbent phase showed excellent specific recognition toward GTFX. Moreover, it was easily separated from the suspension by an external magnet, giving a best selective extraction of drug from biological fluids [21]. 

Many other applications of CNTs in SPE have been performed for the analysis of diverse pesticides (carbofuran, iprobenfos, parathion-methyl, etc.), phenolic preservatives, and natural compounds (piperine from pepper). Moreover, CNTs can be utilized for the extraction of inorganic ions and organometallic compounds as well as for the preparation of stationary phases of GC or LC columns [13, 44].

## 5. Pharmacokinetics and Metabolism of Carbon Nanotubes

Studies in pharmacokinetics and metabolism of diverse forms of CNTs have been investigated and some review articles about them have been reported in the literature recently [2, 8, 45]. The biodistribution and pharmacokinetics of nanoparticles depend on their physicochemical characteristics such as surface functionalization, solubility, shape, aggregation, and chemical composition. In the literature, two studies were performed with water soluble CNTs (SWCNT or/and MWCNT) for their biodistribution in mice [2, 45–47]. None of these studies reports toxic side effects or mortality. Both experiments have used either ^125^Iodine or ^111^Indium as radio tracers for observing their biodistribution in mice [46, 47]. In the first one, ^125^I-hydroxylated-SWCNT (^125^I-SWCNT-OH) was administrated by different routes (intraperitoneal, IV, SC, or oral). This study reported that the CNT biodistribution was not significantly influenced by the administration route and that the ^125^I-SWCNT-OH distributed quickly throughout the whole body with 94% of the unchanged nanotubes excreted into the urine and 6% in the feces [2, 46]. The preferred organs for accumulation were the stomach, kidneys, and bone. No tissue damage or distress was reported. Second study used two forms of ^111^Indium-functionalized SWCNT or MWCNT by IV administration only in mice. The biodistribution profiles obtained were found very similar for both types of functionalized CNTs which showed an affinity for kidneys, muscle, skin, bone, and blood 30 min after administration [45, 47]. However, all types of CNTs were rapidly cleared from all tissues and a maximum blood circulation half-life of 3.5 h was determined [2]. Both SWCNT and MWCNT were found to be excreted through the renal route and observed to be intact in the excreted urine by transmission electron microscopy [45].

Nevertheless, some scientists have recently shown that CNTs can be broken down by myeloperoxidase (MPO), an enzyme found in neutrophils of mice [48]. Their discoveries contradict what was previously believed, that CNTs are not broken down in the body. This action of how MPO converts CNTs into water and carbon dioxide can be significant to medicine and thus represents a breakthrough in nanotechnology and nanotoxicology, since it clearly shows that endogenous MPO can break down CNT [3]. 

## 6. Toxicity of Carbon Nanotubes

The results of CNT toxicological assays found in the literature seem to be contradictory. Some preliminary in vitro tests have showed that CNTs are toxicologically benign to certain cells, while other further studies have indicated that CNTs, especially raw materials are potentially dangerous to many living systems [26, 27, 45, 49–64]. It is notified that pharmacological activities of CNTs conjugated with therapeutic molecules are still not applied in men and consequently their clinical toxicity is also not evaluated.

### 6.1. In Vitro Toxicological Studies of Carbon Nanotubes

Some in vitro cytotoxicity assessments of water dispersible single-walled carbon nanotubes (SWCNT) on A549 cells, a human lung cell line, confirmed that there was no intracellular localization of SWCNT in A549 cells and demonstrated that SWCNT could induce an indirect cytotoxicity by alteration of cell culture medium which caused a false-positive toxic effect [49–51]. Dumortier et al. [52] observed that water soluble SWCNTs marked with fluorescein were nontoxic to cultures of mouse B- and T-lymphocytes and macrophages and preserved the function of these immune cells. Other studies aimed to evaluate the potential toxicity and the general mechanism involved in two diameter kinds of water soluble multiwalled carbon nanotubes (MWCNT)-induced cytotoxicity in C6 rat glioma cell line [53]. Results demonstrated that smaller size of MWCNT seemed to be more toxic than larger one. MWCNT-induced cytotoxicity in C6 rat glioma cells was probably due to the increased oxidative stress. However, pristine, water insoluble CNTs, have been found to be highly toxic in vitro to many different types of cells, including human keratinocytes, rat brain neuronal cells, human embryonic kidney cells, and human lung cancer cells [45, 54–56]. It is notified that these insoluble pristine CNTs cannot be used as carriers for drug and gene delivery in therapeutics, but are only found in the workplace of CNT production. 

### 6.2. In Vivo Toxicological Studies of Carbon Nanotubes

According to the interesting review article about CNT toxicity recently published by Yang et al., [45] many in vivo toxicological assessments have been performed by IV or SC injections and gastrointestinal exposure with functionalized or dispersed SWCNTs or/and MWCNTs in different animals (rats, mice). The available safety data collectively indicate that CNTs are of low toxicity via various exposure pathways for biomedical applications. CNTs induced meaningful toxicity only when a very high dosage (60 mg/kg) under Polyethylene-Glycol-MWCNTs (PEG-MWCNTs) form was administrated in mice [45, 57]. The toxicity of SWCNTs is closely related to the oxidative stress in despite of the administration routes [58]. On the other hand, when CNTs have been used as tissue engineering materials for cell growth by implanting subcutaneously, CNTs exhibited very good biocompatibility and did not arise any serious toxicity, except very limited inflammation [59]. However, Folkmann et al. [58] reported that SWCNTs can induce oxidative damages to DNA in mice after oral gavage and Fraczek et al. [59] found that implanted SWCNTs and MWCNTs induced inflammation. In contrast, other reports on the toxicity of CNTs to skin suggested that CNTs were biocompatible to skin with good biocompatibility after subcutaneously planting [45]. For the carcinogenicity evaluations, Takanashi et al. [61] also observed that MWCNTs injected into the subcutaneous tissue of rasH2 mice did not develop neoplasms. However, other scientists found that CNTs introduced into the abdominal cavity of mice showed asbestos-like pathogenicity [54]. Indeed, there are several parameters affecting the toxicity of CNTs in vivo. Metal impurities might contribute partially to the oxidative stress and thus, careful purification of CNTs is necessary. Chemically functionalized CNTs show higher biocompatibility than pristine CNTs. Thus, Yang et al. [45] concluded that functionalized CNTs are generally biocompatible and low toxic for the biomedical purposes. More toxicity evaluations are encouraged to give the safety threshold value of different CNTs and clarify the toxicological mechanism. 

### 6.3. Human Toxicity of Carbon Nanotubes

As applications of functionalized CNTs linked with therapeutic molecules are still not assayed in man for clinical studies, most publications found in the literature suggested that pristine CNTs could be the source of occupational lung diseases in workers of CNT industries like asbestos pathology previously observed in man [45, 60, 62]. Based on several rodent studies in which test dusts were administered intratracheally or intrapharyngeally to assess the pulmonary toxicity of manufactured CNTs, these authors concluded that CNTs were capable of producing inflammation, epithelioid granulomas, fibrosis, and biochemical changes in the lungs [54–56, 60–63]. In a recent publication in 2013 [64], Ali-Boucetta et al. reported that the asbestos-like reactivity and pathogenicity reported for long, pristine nanotubes can be completely alleviated if their surface is modified and their effective length is reduced as a result of chemical treatment, such as with tri(ethylene glycol) (TEG). However, opinions about the potential hazards of exposures to pristine CNTs and their residual metal impurities are still discussed. Moreover, the apparent similarity between multi-walled carbon nanotubes (MW-CNTs) and asbestos fibers has raised many questions about their safety profile. Despite their immense potential benefits in therapeutics, the toxicity of CNTs is a major concern that needs to be more clearly explained and apprehended. 

## 7. Conclusion and Perspectives

This minireview reveals many spectacular benefits of carbon nanotubes during their recent applications in different areas of pharmacy and medicine. The discovery of this bionanotechnology has opened new alternatives more effective than the ancient drug delivery methods since CNTs can pass through cell membranes, carrying drugs, genes, biomolecules, vaccines, and so forth deep into the target cells or organs previously unreachable. Another novel approach is the use of collagen CNTs materials as scaffolds in tissue generation and artificial implants because CNTs resist biodegradation and are a powerful engineering candidate over other existing materials used to repair defective organs. Besides, CNTs combined with biosensors or other materials have proven excellent implements for the therapeutic monitoring and the diagnosis of diseases as well as for the analysis of drugs in different areas. It is also advisable to develop the free radical scavenger potential of functionalized CNTs for health maintenance. Overall, this nanotechnology could revolutionize the therapeutic concepts in the future and give a glimmer of hope for the treatment of many incurable diseases.

However, despite many surprising results of CNTs obtained during the beginning of this research field, there are still tremendous opportunities to be explored and significant challenges and risks to be solved. Therefore, more imagination and innovation are needed to elaborate different new forms of CNTs and their conjugates with high efficacy and safety for medicinal use in the future. For example, future preparation of CNTs should be attached with new sensitive markers so that they could directly reach the target cells or scientists could easily drive them from the exterior to the target organ in order to avoid side effects on the other healthy tissues. 

In the same time, many hurdles of this nanotechnology have to be solved or elucidated. The human toxicity of different forms of CNTs for short- and long-term treatment is the first vital concern when they will be assayed in clinic. Some preliminary toxicological studies in vitro and in animal that were recently performed have shown controversial results. Carefully optimizing the physicochemical parameters to minimize the toxicity of CNTs is highly favorable. More toxicological investigations of different forms of CNTs from pristine CNTs to functionalized CNTs and their conjugates are highly recommended before they could be effectively used in clinical study and then marketed worldwide.

## Figures and Tables

**Table 1:** Comparison between single-walled carbon nanotubes (SWCNT) (A) and multi-walled carbon nanotubes (MWCNT) (B) [3].

SWCNT	MWCNT
(1) Single layer of graphene	Multiple layer of graphene
(2) Catalyst is required for synthesis	Can be produced without catalyst
(3) Bulk synthesis is difficult	Bulk synthesis is easy
(4) More defection during functionalization	Less defection, but difficult to improve
(5) Purity is poor	Purity is high
(6) Less accumulation in body	More accumulation in body
(7) Easy characterization and evaluation	Difficult characterization and evaluation
(8) Easily twisted	Difficult to twist
